# Variable expressivity of the tumour suppressor protein TRP53 in cryopreserved human blastocysts

**DOI:** 10.1186/1477-7827-5-39

**Published:** 2007-10-17

**Authors:** Vashe Chandrakanthan, Omar Chami, Tomas Stojanov, Chris O'Neill

**Affiliations:** 1 Human Reproduction Unit, Royal North Shore Hospital, St Leonards, NSW, 2065, Australia and the Discipline of Physiology, University of Sydney, Sydney, NSW, Australia; 2 Sydney IVF, 321 Kent St. Sydney, NSW, Australia; 3 Discipline of Medicine, University of Sydney, Sydney, NSW, Australia

## Abstract

In a mouse model, in vitro fertilization or extended embryo culture leads to the increased expression of TRP53 in susceptible embryos. Ablation of the TRP53 gene improved embryo viability indicating that increased expression of TRP53 is a cause of the reduction of embryo viability resulting from in vitro fertilization or embryo culture. This study investigates the status of TRP53 expression in human embryos produced by intracytoplasmic sperm injection. Following fertilization, embryos were cultured for 96 h and then cryopreserved. Immediately upon thawing they were fixed in formaldehyde and subjected to immunostaining for TRP53. Staining was visualized by confocal microscopy. Negative controls were incubated with isotype control immunoglobulin and showed negligible staining. All embryos showed TRP53 staining above negative controls. TRP53 staining was heterogenous within and between embryos. An embryo that showed retarded development showed high levels of TRP53 expression. A blastocyst that had a collapsed blastocoel also showed high levels of TRP53 compared to morphologically normal blastocysts. Most TRP53 staining was in the region of the nucleus. Morphologically normal blastocysts tended to show little nuclear accumulation of stain. However, some cells within these embryos had high levels of nuclear TRP53 expression. The results show that embryos have varying sensitivity to the stresses of production and culture in vitro, and this resulted in variable expressivity of TRP53.

## Background

It is well recognised that a large proportion of embryos produced by in vitro fertilization (IVF) and cultured in vitro do not have the capacity to produce viable offspring. Some studies have examined the patterns of gene expression in human embryos in vitro to determine whether expression of any genes is predictive or informative of the embryo's developmental fate. One such study found an apparent association between the level of expression of transformation related protein 53 (*TRP53*) mRNA and the normality of embryo morphology [1].

*TRP53 *mRNA is expressed in human embryos produced by IVF [1,2]. Its expression was low in oocytes, the zygote and cleavage stage embryos but showed an approximate 10-fold increase in transcript number at the blastocyst stage. A statistically significant association was observed between the degree of fragmentation of cells and the level of *TRP53 *mRNA expression [1], although this association was highly variable. It was suggested that the overexpression of *TRP53 *observed in these fragmenting embryos may infer increased responses by some embryos to genotoxic and/or non-genotoxic stress in vitro [1].

In a mouse model, IVF or extended culture of zygotes resulted in increased expression of TRP53 protein in the resulting blastocysts when assessed by western blot analysis or immunolocalization [3,4]. TRP53 staining in these embryos was higher in the nucleus, and was transcriptionally active, since it leads to the increased expression of BAX [3]. The level of TRP53 expression was related to the rate of development of embryos in vitro and their capacity for ongoing foetal development, while the deletion of *TRP53 *ameliorated some of the adverse effects of embryo culture [3,4]. There is much anecdotal evidence that transgenic over-expression of *Trp53 *is incompatible with early mouse embryo development.

A mouse strain (C57BL/6) that showed high levels of TRP53 following culture had poor embryo viability upon embryo transfer, compared with a strain (hybrid B6CBF1) that showed little TRP53 expression following culture. Furthermore, the increased TRP53 expression that occurs in *MDM2*^-/- ^embryos caused the failure of embryo development and embryonic lethality [5-7]. In another model, diabetes-induced early embryopathy was partially ameliorated by *TRP53 *deletion in mice [8]. These lines of evidence show TRP53 expression may be an important determinant of embryo viability. It was concluded that in the mouse model the increased expression of TRP53 was a consequence of unidentified stressors of IVF and culture and that its expression was one cause of the reduction in viability and developmental potential by some embryos produced by IVF subjected to culture [3,4].

The high levels of *TRP53 *mRNA in some human embryos produced by IVF [1,2] may suggest that expression of TRP53 is also a determinant of human embryo viability. It is noteworthy, however, that TRP53 protein expression is not primarily regulated at the level of transcription. A major component of the regulation of protein levels of TRP53 is by modulation of the half-life of the protein [9]. The MDM2/HDM2 protein binds TRP53 and targets it for ubiquitin-mediated degradation by the cellular proteosome [10,11]. Thus, cellular TRP53 levels are maintained in a latent state by its continual degradation. A range of genotoxic and non-genotoxic stressors act to disrupt MDM2/HDM2 mediated degradation of TRP53, allowing its rapid accumulation within cells subjected to a range of stressors. Thus an increase in mRNA levels may not necessarily predict increased TRP53 protein expression in blastocysts.

This study examined the level and cellular localization of TRP53 expression in human embryos produced by intracytoplasmic sperm injection (ICSI) followed by culture for 4 days. The results show that TRP53 expression is highly variable within and between embryos, with some embryos showing high levels of TRP53 staining. Blastocysts with good morphology tended to show less staining than those that were retarded or of poor morphology. The results of this pilot study show that the expression of TRP53 is highly variable in embryos produced and cultured in vitro.

## Methods

A total of 7 embryos from two couples were examined. Both couples had received treatment for male factor infertility (oligozoospermia; <5 × 10^6^/mL). Embryos were created by ICSI, as previously described [12]. Embryos that were excess to the needs of the treated couples were donated, with informed consent, under NHMRC licence 309702B. All other procedures were as previously described [13]. Embryo culture was carried out in the Sydney IVF sequential media suite (Cook Australia Pty Ltd, Brisbane, Qld, Australia) under low-oxygen conditions (5% oxygen, 6% carbon dioxide, 89% nitrogen); using Nunclon four-well dishes (Nunc, Roskilde, Denmark) in 10 μL drops. On day 3 after fertilization, the embryos were transferred from low-glucose, pyruvate-dominant Sydney IVF Cleavage Medium (zero glucose, 0.2 mmol/L pyruvate) to glucose-dominant Sydney IVF Blastocyst Medium (3 mmol/L glucose, 0.2 mmol/L pyruvate). Under Australian legislation, only excess embryos that have been cryopreserved are available for research. Embryos not immediately transferred were incubated in 5% glycerol (v/v) and then in 9% glycerol (v/v) and 0.2 M sucrose in preparation for cryopreservation (Sydney IVF Blastocyst Freezing Kit; Cook). Embryos were placed in straws (IMV Technologies, France) and loaded into a rate-controlled freezer (Kryo 10 series II; Planar, Middelsex, England). Straws were cooled to -7°C at 2°/min, then -7°C to -30°C at 0.3°/min, and from -30°C to -150°C at 35°/min, then plunged directly into liquid nitrogen at -196°C. Blastocysts were thawed by removing the straw from the liquid nitrogen and exposing the straw to room temperature for 30 sec, followed by 30 sec in a 30°C water bath. The straw was cut open onto a Petri dish and the embryos were then sequentially passed through buffers containing 1.0 M propanediol followed by 0.5 M propanediol (each with 0.2 M sucrose) and then into 0.2 M sucrose (Sydney IVF Blastocyst Thawing Kit; Cook). After washing in Hepes buffered media embryos were immediately fixed in formaldehyde (2%) and subjected to immunolabelling for TRP53.

### Grading of embryos

Upon thawing embryos were assigned a score [14]. Briefly, blastocysts were given a numerical score from 1 to 4 on the basis of their degree of expansion, as follows: 1, an early blastocyst with a blastocoel that is less than half of the volume of the embryo; 2, a blastocyst with a blastocoel that is half of or greater than half of the volume of the embryo; 3, a full blastocyst with a blastocoel completely filling the embryo; 4, an expanded blastocyst with a blastocoel volume larger than that of the early embryo, with a thinning zona. For blastocysts graded as 3 or 4, the development of the inner cell mass was assessed as follows: A, tightly packed, many cells; B, loosely grouped, several cells; or C, very few cells. The trophectoderm was assessed as follows: A, many cells forming a cohesive epithelium; B, few cells forming a loose epithelium; or C, very few large cells. The grade is represented as NXY: where N is blastocyst score; X, inner cell mass score; and Y, trophectoderm score.

### Immunolocalization

Immunolocalization procedures were the same as previously described for mouse embryos [4]. Briefly, embryos were washed 3 times in PBS with 0.1% (w/v) BSA, 0.1% (v/v) Tween-20 and 0.2% (w/v) sodium azide (washing buffer) and then fixed with freshly prepared 2% paraformaldehyde (w/v) (Sigma) in PBS (pH 7.4) for 30 min. This was followed by permeabilization with 2% paraformaldehyde with 0.3% Tween-20 (Sigma) at room temperature for a 30 min. Embryos were washed 3 times in washing solution and then were blocked in PBS containing 2% (w/v) BSA, and 30% (v/v) goat serum for 3h. They were stained overnight at 4°C with primary antibody 1:500 anti-TRP53 (Ab-7) polyclonal antibody (Cat No: PC35, Oncogene Research Products, Nottingham, NG9 2JR, UK) or an equivalent concentration of isotype control immunoglobulin (negative control). Primary antibody was detected by incubation of embryos with secondary antibody coupled to FITC in PBS, 2% BSA for 1 h at room temperature. Optical sectioning was performed with a BioRad Radiance Confocal microscope, using a Nikon Plan Apo 60X/1.4 oil emersion objective. Images were captured using Lasersharp 2000, Version 4.0 (BioRad). Microscope and laser settings were adjusted such that no fluorescence was observed with nonimmune controls. All the test specimens were observed with these same settings.

## Results

**Figure 1:**
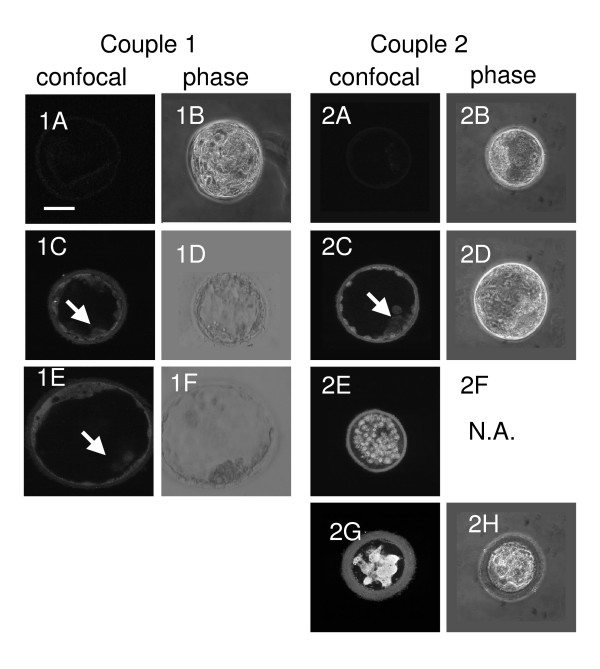
Staining of several embryos from 2 couples (1 & 2) with either nonimmune IgG (1A and 2A) or TRP53 antibody (all other embryos). Two images of each embryo (except 2C) are provided: an equatorial confocal section (confocal) or phase contrast (phase). All confocal images were taken with identical microscope, laser and magnification settings. The position of the inner cell mass is identified by a white arrow where applicable. The bar is 10 μm, N.A. is not available.

**Figure 2:**
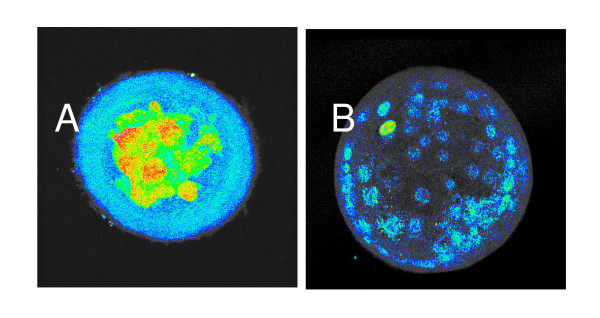
Compilation of sequential z-sections through the entire depth of two embryos shown in Fig 1. A. compilation of sections from embryo shown Fig 1 2G; B. compilation of embryos from Fig1 2C. Greyscale images were converted to pseudocolor representation of staining intensity (blue lowest intensity, red greatest intensity).

The expression of TRP53 in human embryos was assessed by immunofluorescence with confocal microscopy. Staining of 7 human embryos for immunodetectable TRP53 showed considerable within and between embryo heterogeneity of TRP53 expression. Figure 1 shows single equatorial optical sections through each embryo. Nonimmune (control) immunoglobulin samples showed no staining for a blastocyst with some signs of degeneration (embryo grade 3BB) (Fig 1A–B) or in a morphologically normal blastocyst (grade 3AA) (Fig 1, 2A–B). Five embryos were stained for TRP53. Two embryos were graded as 3BA (Fig 1C,D and 2C,D). These blastocyst showed a relatively high levels of staining in some nuclei of the trophectoderm, but this was less apparent in the cells of the inner cell mass (Fig 1C, 2C). One blastocyst showed extensive expansion (grade 4AA) and there was little obvious TRP53 staining in this blastocyst (Fig 1E–F). One blastocyst had either failed to expand or had collapsed (grade 1) (Fig 1, 2E), and it displayed obvious nuclear accumulation of TRP53 staining in all cells. In a blastocyst with obvious signs of degeneration (grade < 1) (Fig 1, 2G,H) there was extensive TRP53 staining throughout the embryo.

The embryos shown in Fig 1, 2G and 2C were reconstructed by compilation of all z-sections through the entire embryo shown in (Fig 2A and 2B, respectively). The images were transformed by pseudocolor representation of staining of TRP53 intensity. Figure 2A confirms the extensive staining throughout all cells and shows some staining in the perivitelline space and zona pellucida in the degenerating embryo. Figure 2B shows a small number of cells expressed high levels of TRP53 within the nuclear region in an otherwise morphologically normal blastocyst (grade 3BA). There was little evidence of significant staining within the inner cell mass, while some cells within the trophectoderm showed marked levels of nuclear staining.

The results show that human embryos in culture display variable levels of TRP53 staining, and this variability existed both within and between embryos. While embryos of poorest morphology showed the highest level of TRP53 staining, the level of expression within morphologically normal blastocysts was not predicted by the embryo's grade.

## Discussion

TRP53 is a transcription factor that induces the expression of a complex transcriptome. It has roles in the regulation of the rate of cell-cycle progression and the survival or death of cells [9,11,15]. TRP53 also has a range of actions that are independent of its transcriptional activities [16]. In normal cells its expression is latent due to its rapid degradation by the proteosome. Changes in the rate of TRP53 degradation can lead to its acute upregulation, and this commonly occurs as a response of the cell to a range of genotoxic and non-genotoxic stressors [11,17].

This study shows that within a sample of human embryos produced by ICSI, two embryos that were morphologically abnormal displayed high levels of TRP53 staining. In these embryos TRP53 staining was most intense in the region of the nucleus. By comparison all embryos that formed morphologically normal blastocysts had lower levels of TRP53 staining. It is noteworthy, however, that there was variability in expression of TRP53 both within and between blastocysts of normal morphology. Thus, some cells in otherwise normal blastocysts showed high levels of TRP53 staining. However, the blastocyst that had the highest rating for morphology (grade 4AA) showed the least TRP53 staining. The high levels of expression of TRP53 observed in embryos of the poorest morphology is consistent with the observation that embryos with poorest morphology expressed the highest levels of *TRP53 *mRNA [1].

It is generally accepted that embryos with retarded development and those with poor morphology are less likely to form viable embryos upon transfer to recipients [14,18]. Given the demonstrated role of TRP53 expression in reducing embryo viability in the mouse model [3,4], the high rates of TRP53 expression in some human embryos may well be a cause of this lower developmental potential. It is not clear from this study whether the expression of the high levels of TRP53 is a cause or consequence of the poor development of these embryos.

It is also generally accepted within human ART practice that many embryos of seemingly normal morphology do not have the capacity for full development. It was therefore of interest to note that some embryos of normal morphology also expressed TRP53. The presence of TRP53 in some cells of these embryos infers that TRP53 expression itself does not necessarily induce acute loss of morphological normality up to the blastocyst stage, and this is consistent with the observations made in mice [3,4]. In a mouse model, inbred mice, which are highly susceptible to the stressors of culture, expressed high levels of TRP53 following culture (even in morphologically normal blastocysts). Yet these blastocysts had low rates of developmental viability following embryo transfer [3]. The genetic deletion of *TRP53 *significantly reversed this loss of viability in cultured embryos [4], arguing that the upregulation of TRP53 expression in culture is a causative agent in loss of the embryo's developmental potential irrespective of embryo morphology.

We choose to examine embryos that were fertilized by ICSI. This was undertaken with the view that oocytes from this population were most likely to be without inherent defects. The process of ICSI itself did not cause increased TRP53 expression, since several embryos fertilized in this manner did not show high levels of expression. It is known that sperm from some men have definable rates of DNA damage [19]. This study does not address whether sperm based defects are a causative agent of the expression of TRP53, yet it is notable that the embryos with highest levels of expression where all from the same couple.

As a consequence of Australian government legislative requirements, it was only possible to examine TRP53 expression in embryos that had first been cryopreserved. This study cannot, therefore, exclude the possibility that some of the variability in TRP53 expression was a consequence of cryopreservation. This seems unlikely since the embryos were fixed immediately upon thawing, and cooling would seem to militate against increase protein synthesis. It is clear that cryopreservation itself was not the cause of increased TRP53 expression since some embryos showed little TRP53 expression despite their cryopreservation. However, we can not exclude this possibility that it contributed to the expression in some embryos, and since this variable could not be controlled for in this study it is important its potential effects be further assessed in jurisdictions that are not so constrained in their use of embryos for research purposes.

## Conclusion

This study shows that some human embryos produced by ICSI and subjected to prolonged culture express the tumour suppressor protein, TRP53. The high degree of variable expressivity of this protein shows that there is considerable variability in the responsiveness of embryos, and some cells within embryos, to the various stressors of ICSI and culture. The study confirms the negative association between the expression of TRP53 and embryo development. The results indicate that in the human embryo, expression of TRP53 may be one of the conditions that determine the viability and developmental potential of the embryo, as is the case in the mouse.

## Competing interests

CO declares that he is an applicant for a patent relating to some of the content of the manuscript.

## Authors' contributions

VC, carried out the immunostaining. OC and TS undertook embryo culture cryopreservation and thawing. CO'N, conceived of the study, and participated in its design and coordination, performed the confocal microscopy and drafted the manuscript. All authors have read and approved the final manuscript.
